# A Clinical Study on the Efficacy and Tolerability of a New Topical Gel and Toothpaste in Patients with Xerostomia: A Randomized Controlled Trial

**DOI:** 10.3390/jcm10235641

**Published:** 2021-11-29

**Authors:** Pia López Jornet, Laureano Hernandez, Francisco Gomez García, Fe Galera Molero, Eduardo Pons-Fuster López, Asta Tvarijonaviciute

**Affiliations:** 1 Faculty of Medicine and Odontology, Biomedical Research Institute (IMIB-Arrixaca) Hospital Morales Meseguer, Clínica Odontológica, Marqués del los Vélez s/n, 30008 Murcia, Spain; pialopezj@gmail.com (L.H.); fjgomez@um.es (F.G.G.); fegaleramolero@gmail.com (F.G.M.); 2 Departamento de Anatomía Humana y Psicobiología, Faculty of Medicine and Odontology, Biomedical Research Institute (IMIB-Arrixaca), University of Murcia, 30100 Murcia, Spain; eduardo.p.f@um.es; 3 Interdisciplinary Laboratory of Clinical Analysis INTERLAB, International Campus Excellence “Campus Mare Nostrum”, University of Murcia, 30100 Murcia, Spain; asta@um.es

**Keywords:** xerostomia, saliva, dry mouth, gel, toothpaste, cytokines

## Abstract

Objective: xerostomia is a very common problem in the general population. The objective of this study was to determine the efficacy of a new gel and toothpaste in patients with xerostomia, analyze the role of salivary cytokines as biomarkers of xerostomia and assess the possible changes in salivary cytokines following treatment. Materials and methods: A randomized, controlled double-blind clinical study was carried out in 73 patients with xerostomia divided into two groups: placebo and active treatment (cymenol; tocopheryl acetate; D-panthenol; Aloe barbadensis; citrate tribasic dihydrate; fluoride) with oral gel and toothpaste three times a day for four consecutive weeks. The Thomson Xerostomia Inventory was applied, with the assessment of oral quality of life (OHIP-14) at baseline and after four weeks of application of the product. Sialometry was also performed in both groups, with analysis of the IL-1b, IL-6, IL-8 and TNFa levels in saliva. Results: In the active treatment group, the xerostomia scores decreased significantly at the end of the study versus baseline, from 33.47 to 27.93 (*p* < 0.001). No significant decrease was recorded in the placebo group (34.5 to 32.75; *p* = 0.190). There were no adverse effects in either group. Regarding the saliva samples, the active treatment group showed significant differences in IL-6 concentration versus the control group (18.55 pg/mL (8–38.28) and 5.83 pg/mL (1.19–12.04), respectively; *p* = 0.002). No significant differences in salivary cytokines were observed in either the treatment group or the control group. Conclusions: The use of a new toothpaste and gel developed for patients with xerostomia proved effective, with greater symptom relief than in the placebo group. Further clinical studies involving longer time periods and larger samples are advisable in order to confirm the benefits of the described treatment.

## 1. Introduction

It has been estimated that xerostomia (dry mouth) affects approximately 20% of the population [1,2,3], with prevalences ranging from 12–39% [2,3,4,5,6]. The disorder is more common among the elderly and in individuals receiving multiple drug treatments [4,5,6] and, moreover, women appear to be more susceptible than men [1,2,3].

Dry mouth is a subjective complaint. The patients affected typically experience problems with chewing, swallowing and even speaking [6,7,8]. While dry mouth is a subjective condition, hyposalivation refers to an objective and, therefore, measurable decrease in salivary flow [8].

The main causal factors of dry mouth are head and neck radiotherapy, systemic diseases, such as Sjögren’s syndrome and the use of certain drugs [7,8,9,10]. Xerostomia complaints are conditioned more by the number of drugs prescribed than by a specific type of drug, i.e., each medication used may exacerbate the dry mouth sensation when combined with other drugs [5]. The literature also supports the idea that xerostomia may be proportionate to the number of medications used [5,6]. In this respect, the main drugs capable of inducing hyposalivation are anticholinergic agents, antidepressants, antihypertensive drugs, antihistamines and sedatives [6], possibly because these substances possess antimuscarinic properties. Although some studies [2,6] have attempted to quantify the effects of different drugs upon the salivary glands, the data obtained are inconclusive due to difficulties in determining the effects of the administered dose, the absorption and excretion rates and the interactions between different drug substances.

The treatment of xerostomia should be established on an individualized basis, with measures that range from simple strategies to stimulate saliva output (chewing gum or sweets) to more complex protocols [10,11,12]. In this regard, the prescription of systemic sialogogues with anticholinesterase and cholinergic action may prove effective, but they are generally avoided because of their numerous side effects in the form of flushing, perspiration or nausea and interactions with other drugs [12,13,14,15,16,17].

In this context, the application of topical sialogogues may be useful for treating dry mouth [10,11,12,13,14,15,16,17,18]. Salivary substitutes may contain substances of natural origin (including salivary macromolecules, such as mucins, lysozyme or lactoferrin) that ensure good biocompatibility. Unfortunately, the data on the efficacy of these formulations are ambiguous [19,20,21]. The existing studies are typically biased by small sample sizes and by methodological shortcomings [11].

A newly formulated mouth treatment contains, among others: cymenol; tocopheryl acetate; D-panthenol; Aloe barbadensis; citrate tribasic dihydrate; and fluoride salivary flow stimulant to provide symptomatic relief from dry mouth symptoms.

Since a broad range of salivary substitutes are available, randomized trials are needed to examine their efficacy in specific patient populations [18,22]. The scarcity of data on the clinical efficacy of salivary substitutes in patients with xerostomia justifies the need for more in-depth investigations.

Cytokines are a group of small proteins involved in the regulation of infection, immune response and inflammation. They also constitute an important presence in saliva, intervening in multiple physiological and disease processes in the oral cavity. In this regard, cytokines play a central role in the initiation and perpetuation of secretory gland inflammation. Imbalances in anti-inflammatory cytokines can result in cumulative salivary gland damage and impaired secretory function [23,24,25,26,27]. Salivary cytokines have been investigated in patients with xerostomia as a secondary condition to Sjögren’s syndrome, with the observation of an increase in salivary interleukin-6 (IL-6) in such individuals—though few studies have been conducted on xerostomia due to other causes [24,25,26]. Improved knowledge of the biological mechanisms involved could possibly contribute to identifying potential therapeutic targets in these patients.

The objective of this study was to determine the efficacy of a new gel and toothpaste versus placebo in relation to subjective xerostomia symptom burden after four weeks of treatment, analyze the role of salivary cytokines as biomarkers of xerostomia and assess the possible changes in salivary cytokines following treatment.

## 2. Materials and Methods 

### 2.1. Participants

The study sample consisted of 73 patients diagnosed with xerostomia and seen in the Department of Oral Medicine (University of Murcia, Murcia, Spain). All of them were receiving medication capable of causing hyposalivation, as reflected by the patient case history.

The prescribed medications included the following: antihypertensive drugs (*n* = 40) (beta-blockers, selective beta-1-blockers), vasodilators, calcium antagonists, angiotensin converting enzyme inhibitors (ACEIs), diuretics (*n* = 10) (furosemide), antihistamines (diphenhydramine) (*n* = 9), antidepressants (*n* = 32) (duloxetine, amitriptyline, clomipramine, escitalopram, fluoxetine, paroxetine), antipsychotics (*n* = 5) (clozapine), bronchodilators (*n* = 9), oxybutynin, solifenacin (which reduces bladder activity (*n* = 3), proton pump inhibitors (*n* = 27), oral antidiabetic drugs (*n* = 14) and non-opiate analgesics (*n* = 15).

The study was carried out in line with the recommendations of the Declaration of Helsinki and was approved by the Ethics Committee of the University of Murcia (ID 240/2019) (ClinicalTrials.gov identifier: NCT04184908). Written informed consent was obtained from all the patients.

The inclusion criteria for participating in the study were: age over 18 years; continuous xerostomia for over three months; and/or altered sialometry findings at baseline (salivary flow < 0.1 mL/min).

The exclusion criteria were: xerostomia as secondary to radiotherapy; patients with decompensated systemic disorders or cognitive problems precluding adhesion to the study protocol; pregnant women; and allergy to any of the study treatment components.

A control group of patients with xerostomia was established during the same period and with the same sociodemographic characteristics identified above and matched for age and gender as xerostomia patients. The control subjects were recruited from the Dental School of the University of Murcia and presented no visible oral lesions or chronic inflammatory disease conditions. Oral health and periodontal status were recorded in both groups of volunteers (active treatment and control). Subjects with significant periodontal disease were excluded from the analysis.

### 2.2. Study Design

This was a randomized, double-blind and placebo-active study with a duration of four weeks that followed the Consort Statement guidelines (http://www.consort-statement.org/, accessed on 3 February 2019).

**Figure 1:**
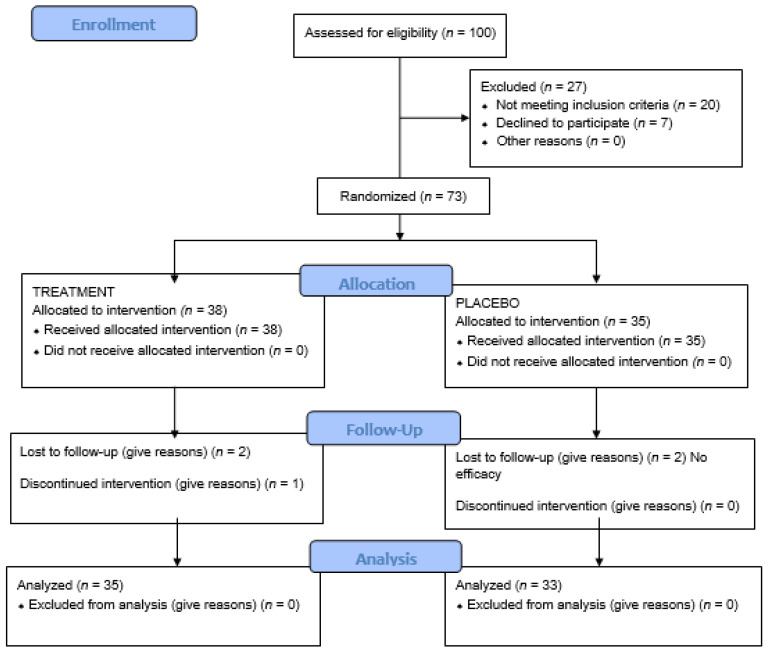
Flow diagram.

Randomization was performed by an external laboratory. The patients and the investigators were blinded to allotment (treatment or placebo group). The study products were coded by an operator unrelated to the study in anonymous opaque containers. The randomization code was kept in a sealed envelope that was not opened until the end of the study. In all cases, the data were compiled by a single investigator blinded to the group to which each individual patient belonged (Figure 1).

### 2.3. Study Products

The company Lacer provided the test and placebo products. The placebo formulation was identical in appearance to the study product but did not contain its active ingredients. Each patient received a kit with the products and instructions for use. The products were to be applied three times a day, and no other gels or oral rinses were allowed for the duration of the study.

The patients were instructed to brush their teeth using the same amount of toothpaste each time (0.5 g). The gel in turn was to be applied after brushing, using clean fingers to distribute 2–3 cm of the product throughout the oral cavity. They were also instructed to avoid eating or drinking for half an hour after the application of the gel. The treatment duration was four weeks. 

The composition of the study products was as follows: 

Xerolacer Boca Seca Gel^®^ (Lacer, Cerdanyola del Vallès, Barcelona, Spain): O-Cymen-5-ol (cymenol) 0.1% (*w*/*w*) (antiseptic; antimicrobial); sodium fluoride 0.064% (*w*/*w*); tocopheryl acetate 0.2% (*w*/*w*) (antioxidant); fluoride ion 500 ppm.

Xerolacer Boca Seca Toothpaste^®^ (Lacer, Cerdanyola del Vallès, Barcelona, Spain): O-Cymen-5-ol (cymenol) 0.1% (*w*/*w*); sodium fluoride 0.32% (*w*/*w*); sodium monofluorophosphate 0.8% (*w*/*w*); dipotassium glycyrrhizate 0.15% (*w*/*w*) (anti-inflammatory); tocopheryl acetate 0.2% (*w*/*w*) (antioxidant); fluoride ion 2500 ppm.

Excipients: D-panthenol (anti-inflammatory); Aloe barbadensis (immunomodulatory and anti-inflammatory); xylitol; sodium hydrogen carbonate; sodium chloride; potassium chloride; monopotassium phosphate; dipotassium phosphate; sodium citrate tribasic dihydrate (salivary flow stimulant).

As commented above, the placebo products and study products had the same appearance and were organoleptically similar. With the exception of fluoride ion (500 ppm in placebo gel and 2500 ppm in placebo toothpaste), the placebo products contained no active ingredients.

### 2.4. Justification of Sample Size 

The level of statistical significance was established as *p* < 0.05, with a statistical power of 80%. The calculated sample size for the comparison of the two groups was 60 subjects (at least 30 in each group). Considering a dropout rate of 15%, at least 70 participants were required (35 in each group).

### 2.5. Collection of Saliva Samples 

Sialometry was performed using the passive droll technique. The sampling was carried out in the morning, and the patients were instructed to avoid smoking, chewing gum or alcohol consumption in the previous one hour. The patients were comfortably seated with the head slightly tilted forwards and with the lips open so that the saliva could drain into a graded tube over a period of 5 min. Sialometry was performed at the start of the study (baseline) and after 30 days, for comparison purposes. Salivary flow was reported in mL/min, and an output of <0.1 mL/min was taken to represent objective hyposalivation. The unstimulated whole saliva was immediately vortexed and centrifuged at 3000 rpm for 15 min at 4 °C, and the clarified supernatant was separated and frozen at −80 °C until further use. Specimens with visible blood traces were discarded.

The study questionnaires described below were all completed in a quiet room without help or interference, and the participants were allowed to take as much time as needed in completing them.

### 2.6. Oral Health Impact Profile (OHIP-14) Questionnaire

This questionnaire, in its short version, was used to detect changes in oral quality of life. The instrument consisted of 14 items that explored different aspects of oral function and quality of life. Higher scores are indicative of poorer oral quality of life [28].

### 2.7. Xerostomia Inventory

The Thomson Xerostomia Inventory assesses the frequency with which patients experience dry mouth, as well as dry skin and conjunctival and nasal dryness. The responses are scored from 0–5, with higher scores indicating a greater severity of xerostomia. The inventory was administered at baseline and again after four weeks [29].

### 2.8. Hospital Anxiety-Depression (HAD) Scale

This scale was used to explore the psychological profile of the patients. It consists of two subscales related to anxiety and depression, respectively. Each subscale in turn comprises 7 items related to disorders of mood state. A score of over 10 indicates the probable presence of anxiety or depression, while scores of ≤7 indicate the absence of significant anxiety or depression [30].

The subjective impression of the patients regarding the administered product was explored based on a score from 0–5 and comprised organoleptic properties, including: flavor and consistency; presentation of the product; convenience of use; interference; efficacy; and whether or not they would be willing to recommend the product.

### 2.9. Study of Salivary Cytokines

The determination of interleukine (IL) 1β, IL-6, IL-8 and tumor necrosis factor alpha (TNFα) in saliva was carried out using the MILLIPLEX MAP Cytokine/Chemokine Magnetic Bead Panel Immunology Multiplex Assay (Millipore, Billerica, MA, USA), following the instructions of the manufacturer. The plates were read with the Luminex 200 system (Millipore). The results were reported in pg/mL.

### 2.10. Statistical Analysis 

Basic descriptive statistics were used. The Student t-test was used to compare means between groups after confirming normal data distribution with the Kolmogorov–Smirnov test and homogeneity of variances with the Levene test.

In order to determine whether the changes in study variables over time were dependent upon the treatment provided, we used two-factor analysis of variance (ANOVA) with repeated measures in one of them, based on the general linear model (GLM), to study the impact of intra- (time: pre–post measures) and inter-subject factors (group: control versus active treatment) upon the dependent variables and their interactions (Group × Time).

The statistical analysis was performed using the SPSS version 25.0 statistical package for MS Windows. Statistical significance was considered as *p* < 0.05.

## 3. Results

A total of 73 subjects with xerostomia were enrolled in the study. Females predominated over males (79.5% versus 20.5%, respectively). The mean age (±standard deviation (SD) was 63.7 ± 12.2 years (range, 35–84 years). A total of 34% were smokers, 56.2% were non-smokers and 9.6% were ex-smokers.

Thirty-eight patients received active treatment (active treatment group) and thirty-five received placebo (placebo group). There were five dropouts in the course of follow-up: three in the active treatment group (two patients underwent systemic medication change, and one had problems in attending the visits) and two in the placebo group (due to a lack of perceived treatment efficacy) (Figure 1).

The control health group in turn consisted of 62 individuals (48 women and 14 men), with a mean age of 59 years. A total of 30% were smokers, 55% were non-smokers and 15% were ex-smokers.

In order to determine the effect of treatment upon the different study variables, we used the two-factor analysis of variance (ANOVA) with repeated measures, based on the general linear model (GLM). Table 1 shows the results corresponding to the intra-subject effects: time (baseline and final measures), group (placebo and treatment) and the interaction of both.

**Figure 2:**
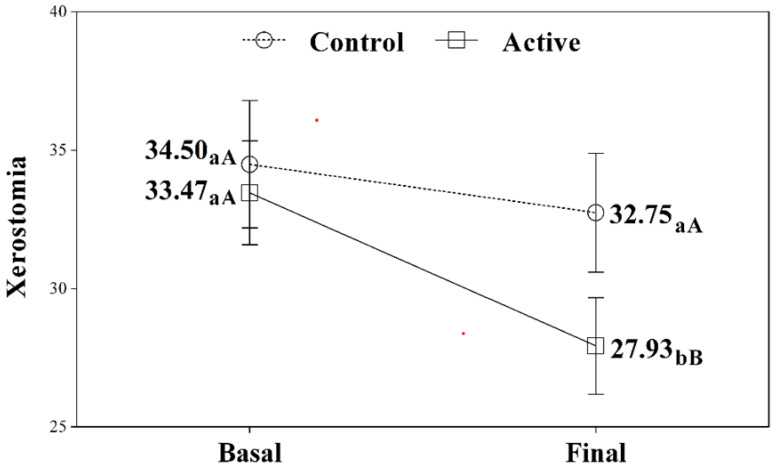
Evolution of xerostomia in the two study groups. Within one same group, the different lowercase letters (a,b) indicate statistically significant differences between the two timepoints (Bonferroni correction). At the same timepoint, different uppercase letters (A,B) indicate statistically significant differences between the two groups (Bonferroni correction).

There was a statistically significant change in the intensity of xerostomia, independent of the group involved (treatment or placebo) (Table 1 and Figure 2). However, the interaction of group and time proved significant, which indicates that the changes in xerostomia score were conditioned by the group to which the individual belonged. Specifically, as regards the Thomson Xerostomia Inventory, the controls showed no statistically significant changes in the final severity score (32.75) versus baseline (34.5) (*p* = 0.190). In contrast, the active treatment group showed a statistically significant decrease of approximately 17% in severity score at the end of the study compared to baseline (*p* < 0.001). At the end of treatment, the intensity of xerostomia among the patients in the active treatment group was significantly lower than in the placebo group (*p* = 0.039).

The anxiety scores were seen to decrease independently of the group involved. Likewise, oral quality of life as assessed by the OHIP-14 improved significantly (*p* = 0.007), regardless of whether the individual belonged to the active treatment group or the placebo group. 

Subjective rating of the products by the patients evidenced high satisfaction scores in both groups in relation to the method of application and organoleptic properties, with no reported adverse effects (Table 2).

### Analysis of Saliva

The analysis of the saliva samples revealed statistically significant differences in IL-6 levels between the active treatment group median (interquartile range) 18.6 pg/mL (8.0–38.3 pg/mL) and the controls 5.8 pg/mL (1.2–12.0 pg/mL) (*p* = 0.002) (Table 3).

No significant differences in salivary cytokines were observed in either the treatment group or the control group.

## 4. Discussion

The high prevalence of xerostomia in the general population has generated interest in the study of this disorder. In many cases, the approach to xerostomia is merely palliative, though only through detailed knowledge of the underlying pathophysiology and of the possible causal agents can we effectively improve its clinical management [2,10,11]. 

The present study has several strengths, including its randomized, placebo-controlled double-blind design. We found the use of a toothpaste and oral gel (three times a day for four weeks) to result in differences between the end of the study versus baseline in both groups—with statistical significance being reached in the active treatment group. The improvement in symptoms of dry mouth was not accompanied by an increase in salivary flow, however. 

At present, over 400 drugs—including many frequently prescribed medications—are known to be able to cause xerostomia. These drugs include antidepressants, antihypertensive drugs, opiates, bronchodilators, proton pump inhibitors, antipsychotics, antihistamines, diuretics, antineoplastic agents and other substances [2,6,8]. The synergic effects of combinations of different drugs also contribute to xerostomia. Although dry mouth induced by drugs is generally reversible, the disease conditions for which such drugs are prescribed are often of a chronic nature [4].

The treatment of xerostomia with systemic sialogogues is generally avoided due to the quantitatively and qualitatively relevant side effects involved [11]. In this context, the use of topical sialogogues may be a useful alternative for treating reversible drug-induced dry mouth, for although the therapeutic effects are shorter lasting compared with systemic treatments, the associated side effects are also less relevant [10]. In addition to humectant and lubricating properties, artificial salivas should contain bioactive ingredients with natural animal or plant extracts to replicate some of the bacteriostatic or antibacterial actions of natural saliva [13,14,15,16,17,18]. The addition of bioactive agents deserves particular attention and is currently probably the most innovative area in the field of artificial salivas. The formulations used in the present study contained antiseptics (cymenol) and mineral salts, and the excipients comprised D-panthenol, Aloe barbadensis, xylitol, sodium hydrogen carbonate, sodium chloride, potassium chloride, monopotassium phosphate, dipotassium phosphate and sodium citrate tribasic dihydrate. The patients allotted to active treatment (toothpaste and gel) experienced improvement of the symptoms, with good tolerance and no adverse effects during the study.

Although a broad range of commercial salivary substitutes are available in gel, liquid, paste, spray, tablet or chewing gum format, the results reported in the literature are contradictory—mainly due to the methodological heterogeneity of the studies involved [11]. Many are open-label studies, while others lack a control group, or the duration of the treatment is very short.

Hahnel et al. [15] conducted a review of artificial salivas in patients subjected to radiotherapy and found such formulations to apparently afford important relief from dry mouth and hyposalivation after radiotherapy. Mucin-based artificial salivas yield better clinical and laboratory test outcomes than saliva substitutes based on carboxymethylcellulose. On the other hand, although mucin affords better results, some patients may be reluctant to use saliva substitutes containing porcine or bovine mucin due to religious or other reasons—a situation that further complicates the choice of an ideal saliva substitute.

With regard to the new types of saliva substitutes in the form of gels, oral rinses and toothpastes, a common complaint is the poor retention of the product within the oral cavity, resulting in a need for frequent applications in order to secure symptom relief [10,11,31,32]. Another problem associated with studies that assess subjective dry mouth sensation is the important placebo effect, and finding an adequate comparator product is also complicated. A double-blind study carried out by Gil-Montoya et al. [33] among institutionalized older persons to assess the effectiveness of an oral gel concluded that only certain aspects of xerostomia are ameliorated and that the placebo effect is very strong and needs to be considered.

Another product for xerostomia relief is Xerostom^®^ [34], which contains olive oil, betaine, fluor and xylitol. Fluor and xylitol control the production of caries, and olive oil is useful for controlling bacterial proliferation. The efficacy of the product has been demonstrated in a controlled clinical trial involving 40 polymedicated patients with dry mouth. A recent study published by López-Pintor [35] has used this same product in a small group of patients with Sjögren’s syndrome, with observable improvement of the symptoms. Well-designed, randomized and controlled trials of sufficient statistical power are needed to analyze topical treatments for dry mouth following the Consort Statement and provide guidelines for clinical management. 

We agree with Barbe et al. [36,37,38] on the importance of the patients’ perspective that providing symptomatic relief is only one part of the whole treatment concept. The treatment of xerostomia should not only include symptom relief, but it should be incorporated into a whole diagnostic, prevention and treatment concept that also addresses the causes of dry mouth and its influence on general health conditions and on oral health and quality of life.

Cytokines, including interleukins (ILs), chemokines, interferons (IFNs) and tumor necrosis factor (TNF), are implicated in the regulation of innate and adaptive immune responses. Cytokines are known to play a mediating role in a range of physiological responses and can contribute to suppression of the production of saliva as seen in natural phenomena, such as aging, and in autoimmune diseases, such as Sjögren’s syndrome [25,26,27]. Altered levels of salivary cytokines IL-6 and TNFa have also been documented in recurrent aphthous ulcers and in oral lichen planus [23,24,25,26,27]. On the other hand, no significant differences in salivary IL-6 and TNFa were observed in patients with burning mouth syndrome versus the control group [27].

The use of saliva as a biological matrix for diagnostic purposes requires the standardization of a number of pre-analytical parameters, such as the sample collection technique employed (i.e., resting or stimulated saliva) and circadian variations. In the present study, all saliva samples were collected in the morning (between 9 and 12 a.m.), in order to standardize the data. The investigations suggest that a multiple approach (e.g., evaluating multiple cytokines at the same time) provides more information than the determination of a single biomarker. Therefore, in the present study we investigated a panel of cytokines in whole saliva among patients with xerostomia, before and after treatment. The findings of our study afford fundamental knowledge for future studies involving larger patient populations. The changes in cytokines did not vary according to the type of intervention involved. We did find the measured cytokine levels to be significantly higher in patients belonging to the active treatment group than among the controls, but these levels were not modified by the treatment provided in either the active treatment group or in the placebo group.

As limitations of our study, mention must be made of the fact that its four-week duration implies that the results might not be representative of the data obtained in the course of years of treatment. We recorded no increase in saliva output, though the symptoms of dry mouth were ameliorated because of treatment. The possible long-term effects, such as dental demineralization and caries, must also be taken into consideration in future studies. On the other hand, limitations should include the limitations of case-control studies (biomarkers), as these studies only provide associations and not a cause–effect relationship.

The increase in life expectancy and advances in life-extending treatments for chronic illnesses are associated with an increase in the number of patients with xerostomia. Dry mouth has an important impact upon the quality of life of these patients. In this regard, topical treatments cause fewer adverse effects and therefore offer greater chances of improving the quality of life and preserving oral health, reducing the risk of additional, painful, weakening and costly oral disorders, as well as the loss of teeth.

In conclusion, the present study suggests that the new oral gel and toothpaste used for four consecutive weeks reduces the severity of xerostomia. Further clinical studies involving longer time periods and larger samples are advisable in order to confirm the benefits of the described treatment.

## Figures and Tables

**Table 1:** Study measurements at baseline and after four weeks of active treatment or placebo.

	Measurement		Intra-Subject Effects	
	Baseline Mean (SD)	Final Mean (SD)	Time	Group Time
			F(df); *p*-Value (eta2)	F(df); *p*-Value (eta2)
SIALOMETRY			F(1;44) = 1.045; *p* = 0.312 (0.023)	F(1;44) = 0.198; *p* = 0.659 (0.004)
Placebo	0.23 (0.20)	0.24 (0.19)		
Active	0.32 (0.25)	0.35 (0.26)		
Total	0.29 (0.23)	0.31 (0.24)		
OHIP-14			F(1;47) = 7.948; *p* = 0.007 (0.145)	F(1;47) = 3.459; *p* = 0.069 (0.069)
Placebo	19.42 (9.86)	18.89 (10.12)		
Active	20.40 (11.86)	17.83 (9.47)		
Total	20.02 (11,03)	18.24 (9.63)		
XEROSTOMIA INVENTORY			F(1;48) = 18.327; *p* < 0.001 (0.276)	F(1;48) = 4.945; *p* = 0.031 (0.093)
Placebo	34.50 (9.77)	32.75 (10.90)		
Active	33.47 (10.59)	27.93 (8.65)		
Total	33.88 (10.18)	29.86 (9.80)		
HAD-Anxiety			F(1;43) = 4.288; *p* = 0.044 (0.091)	F(1;43) = 0.01; *p* = 0.919 (0)
Placebo	10.25 (5.64)	9.69 (5.57)		
Active	9,03 (4.98)	8.41 (4.95)		
Total	9.47 (5.19)	8.87 (5.15)		
HAD-Depression			F(1;41) = 0.271; *p* = 0.605 (0.007)	F(1;41) = 1.311; *p* = 0.259 (0.031)
Placebo	4.87 (4.31)	5.53 (5.33)		
Active	5.46 (5.17)	5.21 (4.93)		
Total	5.26 (4.84)	5.33 (5.01)		

**Table 2:** Descriptive and comparative satisfaction questionnaire findings between treatments.

	Treatment		Difference of Means	Student-*t*-Test	
	Control	Active		t(49)	*p*-Value
ITEM_1	3.67 (0.91)	3.50 (0.94)	0.17	0.631	0.531
ITEM_2	3.43 (0.98)	3.70 (0.75)	−0.27	−1.122	0.267
ITEM_3	3.67 (0.80)	3.67 (0.55)	0.00	0	1
ITEM_4	3.05 (1.32)	3.67 (0.84)	−0.62	−2.042	0.047
ITEM_5	2.25 (1.33)	2.23 (1.41)	0.02	0.042	0.967
ITEM_6	3.37 (0.96)	3.28 (1.07)	0.09	0.306	0.761
ITEM_7	3.50 (0.76)	3.43 (1.01)	0.07	0.252	0.802
ITEM_8	3.60 (0.75)	3.53 (0.90)	0.07	0.273	0.786

Item 1. Do you consider the product presentation in gel format to be adequate? Item 2. Has it been easy and convenient to use this product? Item 3. Does the product have a pleasant taste? Item 4. Was the consistency of the product pleasant? Item 5. Did the taste of the product interfere with that of the foods you eat? Item 6. Do you consider it adequate for improving dry mouth? Item 7. Would you use this product again? Item 8. Would you recommend this product to others?

**Table 3:** Analysis of interleukins and TNFa in patients with xerostomia and the controls.

Analyte	Control	Xerostomia	*p*-Value
IL-1b, pg/ml	25.24 (2.5–105.8)	36.1 (6.425–125.3)	0.517
IL-6, pg/ml	5.83 (1.19–12.04)	18.55 (8–38.28)	0.002
IL-8, pg/ml	728.8 (551.6–1038)	798.4 (402.6–2166)	0.607
TNFa, pg/ml	3.36 (1.51–6.73)	3.55 (1.525–13.88)	0.396

## Data Availability

The data and the statistical analyses can be obtained from the corresponding author upon request.
